# The design of transcription-factor binding sites is affected by combinatorial regulation

**DOI:** 10.1186/gb-2005-6-12-r103

**Published:** 2005-12-02

**Authors:** Yonatan Bilu, Naama Barkai

**Affiliations:** 1 Department of Molecular Genetics, Weizmann Institute of Science, 76100 Rehovot, Israel; 2 Department of Physics of Complex Systems, Weizmann Institute of Science, 76100 Rehovot, Israel

## Abstract

**Background:**

Transcription factors regulate gene expression by binding to specific *cis*-regulatory elements in gene promoters. Although DNA sequences that serve as transcription-factor binding sites have been characterized and associated with the regulation of numerous genes, the principles that govern the design and evolution of such sites are poorly understood.

**Results:**

Using the comprehensive mapping of binding-site locations available in *Saccharomyces cerevisiae*, we examined possible factors that may have an impact on binding-site design. We found that binding sites tend to be shorter and fuzzier when they appear in promoter regions that bind multiple transcription factors. We further found that essential genes bind relatively fewer transcription factors, as do divergent promoters. We provide evidence that novel binding sites tend to appear in specific promoters that are already associated with multiple sites.

**Conclusion:**

Two principal models may account for the observed correlations. First, it may be that the interaction between multiple factors compensates for the decreased specificity of each specific binding sequence. In such a scenario, binding-site fuzziness is a consequence of the presence of multiple binding sites. Second, binding sites may tend to appear in promoter regions that are subject to low selective pressure, which also allows for fuzzier motifs. The latter possibility may account for the relatively low number of binding sites found in promoters of essential genes and in divergent promoters.

## Abstract 2

A comprehensive analysis of binding site locations in yeast shows that essential genes bind selectively few transcription factors and that novel binding sites tend to appear in promoters that are already associated with multiple sites.

## Background

Gene expression is controlled through the action of transcription factors, which bind specific DNA sequences in the upstream region of genes and interact with the basic transcription machinery to facilitate or repress transcription. Characterizing the DNA sequences that serve as transcription factor binding sites is an important first step toward elucidating the logic of transcription regulation. Indeed, advances in experimental and computational methods generated a genome-wide mapping of *cis-*regulatory elements in certain model organisms, most notably the budding yeast *Saccharomyces cerevisiae*. In contrast, the principles that govern the design and evolution of such sites are still poorly understood.

For example, it is not clear what controls the length or specificity of *cis-*regulatory elements. These two properties appear to differ greatly between bacteria and eukaryotes; in *Escherichia coli *the average length of a consensus motif is 24.5 base pairs (bp) [1], whereas the average motif length in the fruit fly *Drosophila *is only 12.5 bp [2]. Similarly, whereas the major sigma factor binding-site in *E. coli *has 12 conserved positions [3], the analogous TATA box in eukaryotes is only 6 bp long [4]. Large differences in length also appear for binding sites within the same genome. For example, in *Drosophila *engrailed binds a sequence of 7 bp whereas Adf-1 binds a 21 bp sequence.

Differences in binding-site length may reflect different strategies for maintaining specificity and controlling for random appearances of motifs in unregulated regions. For example, the expected number of randomly appearing sequences of length 24 bp in the *E. coli *genome is about 3.5 × 10^-7 ^(assuming uniform nucleotide distribution). In contrast, spurious appearances of short binding sites are abundant in the large genome of multicellular eukaryotes. In fact, in eukaryotes most apparent binding sites appearances are not functional.

Sequences that are short or 'fuzzy' (that is, far from the so-called consensus motif) can still activate the transcription of certain genes [5]. Specificity in this case requires the combinatorial action of several transcription factors. Indeed, whereas bacterial transcription is typically controlled by a single transcription factor [6], combinatorial regulation is copious in eukaryotes, in which promoters containing 10-50 binding sites for 5-15 different transcription factors are not unusual [7]. However, a direct link between combinatorial regulation and binding-site specificity within the same organism has not yet been demonstrated.

In the present study we used comprehensive mapping of transcription factor binding sites in *S. cerevisiae *to address, on a genome-wide scale, the connection between the length or specificity of a binding site and the degree to which it participates in combinatorial regulation. We further characterized the genes whose regulation involves a large number of binding sites, and the gene promoters that are most amenable to the addition or deletion of binding sites. Based on this analysis, we suggest that multiple occurrences of binding sites within a promoter often reflect weaker negative selection on these regions, allowing for the accretion of binding sites.

## Results

### The number of binding sites is correlated with expression variability

To examine whether there is a connection between combinatorial regulation and the length of transcription factor binding sites, we considered the comprehensive map of *S. cerevisiae *binding site locations, derived by Harbison and coworkers [8]. This map was generated using a ChIP-chip assay, characterizing all promoter regions that bind a specific transcription factor, followed by a computational analysis that predicted the precise location of each binding site. All together, the data set includes 9,715 binding sites for 102 transcription factors (about 30% of all putative factors), distributed among 2,928 gene promoters.

**Figure 1:**
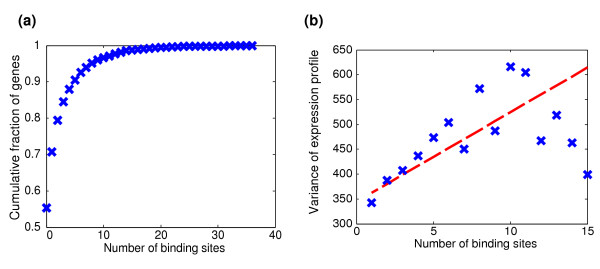
Distribution of binding sites numbers and correlation to gene expression. **(a) **Cumulative fraction of genes according the number of binding sites in their promoter region. **(b) **Expression variance averaged over all genes with like number of binding sites in their promoter. The dashed red line shows the best linear fit to the data points.

The number of binding sites varied greatly among gene promoters. Whereas in most promoters at most one or two binding sites were identified, a fraction of genes (about 4%) exhibited more than ten binding sites in their promoter region (Figure 1a). Genes displaying multiple binding sites in their promoter exhibit a more variable expression pattern (Figure 1b; see Materials and methods, below), suggesting that the number of binding sites appearing in a gene's promoter can serve as a plausible measure of the degree of combinatorial regulation.

### Binding sites for specific transcription factors are less specific when they act in combination with other sites

To examine whether binding site properties depend on their co-appearance with additional sites in the same promoter region, we focused first on binding sites for specific transcription factors. The factor that binds the largest number of genes (293) is Reb1, whose well defined consensus binding site consists of seven nucleotides. As expected, in most gene promoters the predicted Reb1 binding site somewhat deviates from the precise consensus. We considered whether this deviation depends on the number of additional binding sites appearing in the same promoter.

**Figure 2:**
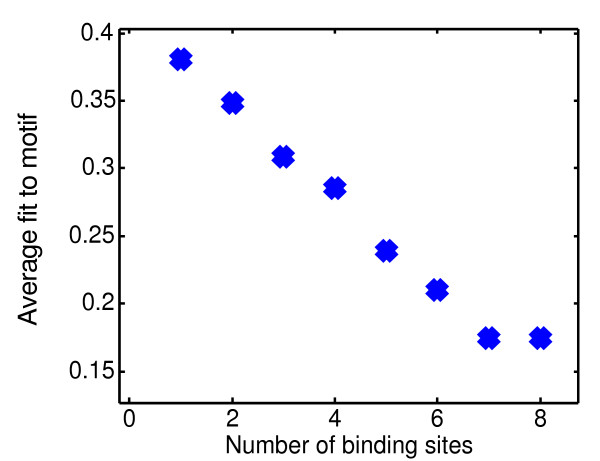
'Fuzziness' of Reb1 binding sites. Average fit of Reb1 binding sites to the consensus matrix, as a function of the number of binding sites within the promoter they appear in.

The match of the Reb1 binding site to its consensus motif decreased sharply with the number of co-appearing binding sites (Figure 2). Although this is particularly striking for Reb1, similar behavior was observed for two-thirds of all 102 transcription factors and for 82.5% of the 40 transcription factors that regulate at least 50 genes (*P *= 5 × 10^-5 ^was estimated for this number of factors, by randomly shuffling the binding sites of each factor and assuming a normal distribution). We conclude that binding sites for a specific transcription factor tend to be less specific when they co-appear with additional binding sites in the same promoter regions.

Because different factors often compete for the same binding site [9], we considered whether the reduced precision of the motif reflects the need to comply with several factors, and perhaps also to tune the binding equilibrium between them. However, our analysis does not support this possibility because there was no significant difference between the fit to the consensus of binding sites that overlap other binding sites and of those that do not. In fact, for 25 of the 40 transcription factors that regulate at least 50 genes, the average fit to the motif was higher for binding sites that overlap other sites as compared with those that do not (see Materials and methods, below).

### Binding sites that appear in combination with other sites tend to be shorter and less specific

**Figure 3:**
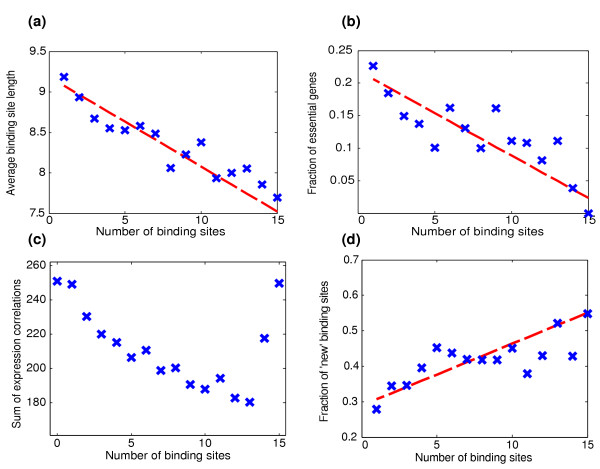
Average promoter and gene properties as a function of the number of binding sites. **(a) **Average binding site length. **(b) **Fraction of essential genes. **(c) **Sum of expression correlations. **(d) **Fraction of binding sites that are 'new' (not conserved in other species). *P *values for the displayed correlations are as follows: (a), 10^-42^; (b), 6 × 10^-7^; (c), 10^-16^; and (d), 10^-22^. Dashed red lines show the linear line that best matches the data points. Graphs show promoters of up to 15 binding sites. These constitute 97% of the promoters for which data are available.

The results above focus on a particular binding site and compare its sequence in different promoter regions. We then considered whether binding sites that tend to appear in promoters containing multiple sites are shorter, on average, than are binding sites that act in isolation. To examine this, we counted for each gene the number of binding sites in its promoter and measured their average length (as it appears in [8]). Indeed, there is a clear inverse correlation between these two values; the higher the number of binding site, the shorter is their average length (Figure 3a; Additional data file 7). Note that length here is defined according to the motif consensus, as indicated by Harbison and coworkers [8].

One possibility is that this negative correlation merely reflects the fact that shorter binding sites appear more often (or are predicted more often by the computational method used). To control for this possibility, we examined the distribution of correlations obtained by reshuffling the binding data. Indeed, the observed correlation is 13.6 standard deviations away from the mean of this random distribution, corresponding to a *P *value of about 10^-42 ^(assuming a normal distribution). Moreover, essentially the same results are obtained when controlling for multiple appearance of the same binding sites, and considering only the number of transcription factors that bind the promoter (Additional data file 4). In contrast to the total number of binding sites, this latter measure is independent of the computational methods used by Harbison and coworkers [8] in defining binding sites.

Importantly, the negative correlation between the length of a binding site and the number of additional sites appearing in the same promoter region does not depend on the precise definition of binding-site length. In fact, similar correlations, with equivalent statistical significance, were observed also for more refined definitions of binding-site length or 'fuzziness', including Euclidean or KL distance of the motif from the background distribution, the average fit of a binding site to the motif, and the probability of a given binding site to appear at random (see Materials and methods, below; also see Additional data file 1).

Particularly informative is the fuzziness measure, which describes the average fit of the motif to its consensus site (Additional data file 1 [panel d]). Longer motifs are expected to have more ambiguous positions than shorter ones because there is some flexibility in defining the boundaries of a binding site, and also simply because there are more positions that can be ambiguous. Indeed, when considering all appearances, longer sites tend to be fuzzier than shorter ones (Additional data file 2). Because motif length is negatively correlated with the number of co-appearing sites (Figure 3a), the null hypothesis is that motif fuzziness is negatively correlated with the number of co-appearing sites. The observation that the opposite phenomenon occurs (Additional data file 1 [panel d]) further emphasizes the statistical significance of the correlation between motif fuzziness and the number of co-appearing binding sites.

### Functional characterization of genes under combinatorial control

Taken together, our results suggest that multiple binding sites are associated with shorter and less specific binding sequences. One possibility is that motif multiplicity allows for mutations that decrease the length and specificity of the motif. In this model, interactions between factors can compensate for the decreased specificity of each individual site, ensuring precise expression of the associated gene.

Alternatively, shorter and fuzzier motifs may indicate lower pressure to maintain precise control of the expression of the associated gene. Lower selective pressure would allow for mutations that reduce binding-site specificity on the one hand, and would also allow for the addition of new binding sites on the other. In this case, both binding-site fuzziness and combinatorial regulation reflect the same gene property, but they do not cause each other.

To try to differentiate between the two possibilities, we examined the properties of genes with promoters that exhibit a large number of binding sites. Interestingly, we found that essential genes (in rich glucose medium [10]) are over-represented among genes with few binding sites (Figure 3b). This preferential appearance of binding sites in the promoter regions of nonessential genes, the regulation of many of which we conjecture to be under lower negative selection, supports the possibility that binding site abundance depends on the selective pressure acting on the region.

Genes that are not essential for growth in rich glucose medium might still be essential for growth in other conditions. To complement the analysis described above, we also analyzed the number of binding sites upstream from genes whose knockout led to slow and fast growth in different growth mediums (Yeast Deletion Project [11,12]). As shown in Table 1, in all five conditions for which data are available those genes whose deletion leads to slow growth and whose regulation we conjecture to be under stronger negative selection have, on average, few binding sites. Similarly, genes whose deletion does not hamper growth tend to have a large number of binding sites. We note, however, that these additional conditions are still only a subset of those that are of relevance, and ultimately more experiments are needed to test this hypothesis in full.

As another indicator of the functional importance of the transcriptional regulation of a particular gene, we considered the number of genes that are correlated with it. Indeed, genes that are part of large co-regulated groups tend to exhibit a lower number of binding sites in their promoter region, as compared with genes that are co-regulated with only a few genes (Figure 3c; *P *= 10^-16^). A similar although less significant (*P *= 0.04) correlation was observed for genes that participate in large protein complexes [13].

**Figure 4:**
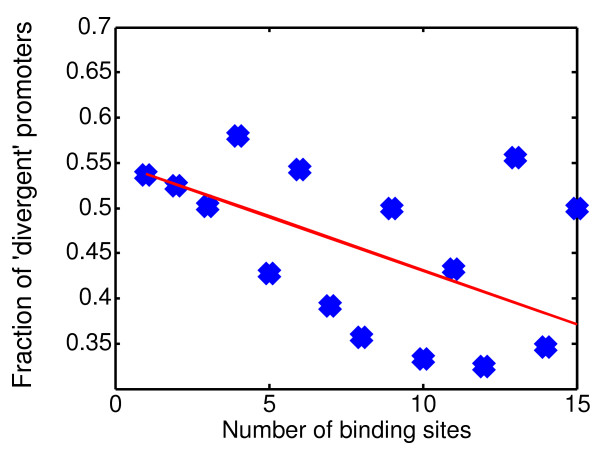
Distribution of 'divergent' promoters. The fraction of promoters that potentially regulate two genes in each subset of promoters with an equal number of binding sites.

The gene properties above provide only an indirect indication of the functional importance of a gene and thus of the selective pressure to maintain its expression. Perhaps a more direct way to identify promoters that are under negative selective pressure is to differentiate between promoters that potentially regulate two genes on the two opposing strands ('divergent promoters') and those that regulate only one. The former group is likely to be under stronger negative selection because mutations there will potentially effect the regulation of both genes. Indeed, as can be seen in Figure 4, divergent promoters tend to exhibit a lower number of binding sites, supporting the proposal that binding site multiplicity reflects lower selection pressure on promoter regions.

Finally, we also looked for Gene Ontology terms associated with sets of genes whose promoters exhibit an exceptionally high or low average number of binding sites (Table 2). Genes involved in metabolism appear to have a higher number of binding sites, but this enrichment is only marginally significant (*P *values shown are the probability for a set of this size to have the observed average number of binding sites).

### 'Preferential attachment' pattern for the addition of new binding sites

Our findings are consistent with a model whereby increased fuzziness and increased number of binding sites both reflect reduced selection pressure to maintain precise expression. To examine this possibility from a different angle, we considered whether new binding sites tend to appear preferentially in some promoter regions. If multiple sites merely compensate for binding-site specificity, then no specific trend is expected. By contrast, if multiple sites (and the fuzziness of binding sites) reflect reduced constraints on gene expression control, then new binding sites would be expected to appear in promoters of genes that already exhibit a large number of binding sites. Indeed, their appearance in such regions is probably less likely to be selected against.

To examine the appearance of new binding sites, we used the data comparing the conservation of binding sites between *S. cerevisiae *and the three *sensu stricto *species whose genomes were recently sequenced [14]. It is likely that sites that are conserved in these species were also present in the genome of the common ancestor and thus represent ancient binding sites. In contrast, binding sites that are not conserved in any of the species may represent the new additions to the *S. cerevisiae *genome.

We found that new binding sites tend to appear in promoter regions that already contain a large number of binding sites (Figure 3d). By randomly shuffling the binding-site data, we estimated this observation to be highly significant (*P *is approximately 10^-22^, assuming a normal distribution).

## Discussion

Specific regulation of gene expression can be realized either by employing a small number of transcription factors with long, unambiguous binding sites, or by employing a larger number of factors, with short, fuzzy motifs. The strategy for transcription regulation in *E. coli *represents one extreme of this approach - most genes are regulated by only one or two transcription factors [6]. On the other extreme are multicellular eukaryotes, whose promoter regions tend to be long and contain many short transcription factor binding sites [7].

Combinatorial regulation is certainly more likely to evolve in species in which binding sites are short and fuzzy, precisely because spurious appearances will occur relatively frequently [15]. Moreover, it might be required because of the greater complexity of eukaryotes [16,17]. Motif fuzziness may be explained by the type of regulation required, for instance when several transcription factors bind the promoter region, and the required logic is that of an AND gate (as in the enhanceosome of interferon-ß in humans [4]). The low affinity for each factor ensures that it initiates transcription only in combination with the other factors and not by itself. In addition, the motif fuzziness might have to do with the fact that in eukaryotes many transcription factors are enhancers, which have less stringent constraints on their appearance [5].

In this work, which focuses on binding site organization within a single organism, we suggest that fuzziness and co-appearance of binding sites may also indicate lower selection pressure to maintain a precise expression pattern of these genes. We provided three pieces of evidence that support this possibility. First, we found a lower level of combinatorial regulation for essential genes and for genes that are part of a large co-expressed module. It is likely that the expression of these genes is more tightly controlled. Similarly, promoters that potentially control two genes ('divergent promoter'), which are also expected to be under stricter selection, tend to have fewer binding sites as well. In addition, we found that new binding sites tend to appear in promoters of genes that already contain a large number of binding sites. Taken together, these results suggest that gene functionality affects the probability that a new binding site will evolve.

A conservative interpretation of this claim is that new binding sites will appear at random where they are not selected against, allowing them the time to evolve toward a more advantageous combination that will lead to specific regulation for different conditions. Alternatively, such stochastic accretion of binding sites may be taken to support previous observations of fitness-neutral variation in binding sites patterns [18,19], and theoretical models for motif fuzziness [20] and position of transcription initiation sites [21]. It might also provide insight to the actual mechanism that allows promoter sequences to evolve, within the context of theories for neutral evolution of gene expression [22,23].

Interestingly, our observation that multiple binding sites are associated with a more variable gene expression profile is explained differently by these two models. In the first it is interpreted as indicating that the gene's expression is tightly regulated, resulting in widely varying levels under varying conditions. In the latter the variable expression profile of many genes is interpreted as being 'fuzzy', due to multiple, nonprecise binding sites. A key goal in distinguishing between these two possibilities is therefore to determine whether expression of genes with multiple binding sites is tightly controlled or, rather, very 'noisy'. With availability of the full library of green fluorescent protein tagged yeast proteins, this can now be tested directly.

## Materials and methods

### Map of transcription factor binding sites

Harbison and coworkers [8] compiled a list of 9,715 binding sites for 102 transcription factors along the *S. cerevisiae *genome. This is largely based on ChIP-chip data, in which binding was determined with high confidence (*P *< 0.001). An array of computational methods was then employed to determine the exact location of each binding site. In addition, the conservation of each site is reported, that is, the number of *sensu strictu *strains (*S. paradoxus, S. mikatae*, and *S. bayanus*) in which it appears. About half the binding sites (51.2%) were found to be conserved in at least two other species.

We define the promoter region of a gene as the 1,000 bp upstream of its translation start site, as listed in the *Saccharomyces *Genome Database [24]. Under this definition, for 2,928 genes there is at least one relevant binding site listed in the dataset. Figure 1a shows the distribution of the number of binding sites among promoter regions.

### Binding-site motifs

Based on the discovered binding sites, Harbison and coworkers [8] constructed, for each transcription factor, a probability matrix for the motif it binds. For a motif of length l, this is a 4-by-l non-negative matrix, in which each column describes the nucleotide distribution in the corresponding position (for example, the sum of each column is 1).

The length of motifs ranges from 5 bp to 19 bp, and the average length in this dataset is 9.3 bp.

### Expression data

Ihmels and coworkers [25] compiled a dataset of 1,011 expression profiles; for each gene and each of 1,011 experimental conditions it lists the log ratio between the observed expression level and the control level. The data were compiled from about 200 environmental stresses conditions, about 100 cell cycle conditions, about 100 sporulation time points, about 300 deletion mutants, about 50 mating-related conditions, and several others.

We define the expression variability of each gene as the sum of squares of these values. This can be thought of as the variance of the log ratio, if we expect the mean to be zero (expression level in experimental condition = control level). We define the level of co-regulation of two genes as the normalized inner product of their expression profiles.

### Essential genes

Giaever and coworkers [10] compiled a list of 1,100 genes that were found to be essential for growth via single knockout experiments. Of these, 505 have at least one binding site in their promoter region, as per the definition given above.

### Growth rates

The Yeast Deletion Project [12] lists relative growth rates for 4,706 homozygous diploid deletion strains, in five different growth mediums: YPD (2% glucose), YPDGE (0.1% glucose, 3% glycerol, and 2% ethanol), YPE (2% ethanol), YPG (3% glycerol), and YPL (2% lactate). We defined 'slow growers' as those strains whose growth rate is at most 75% of wild-type in both reported time courses, and 'fast growers' as those whose growth rate is at least 95% of wild-type in both time courses.

Table 1 lists the average number of binding sites for genes whose deletion leads to slow and fast growth. *P *values were estimated by drawing, at random, subsets of genes of equal size to those listed, and computing the standard deviation of the average number of binding sites over such subsets. From these, Z scores were computed for the real data, and the *P *values were estimated assuming a normal distribution.

### Measures of fuzziness

We suggest four ways to measure the fuzziness of a binding site or of a motif. The first two methods can be thought of as refinements to simply looking at the length of a motif. The third and fourth measure fuzziness more directly:

#### Euclidean distance from background

A motif of length *l *is represented by a 4-by-*l *matrix M (as described under Binding-site motifs, above). Let *B *be the 4-by-*l *matrix corresponding to the background distribution; that is, each column contains the overall nucleotide frequency (31% for A and T, 19% for C and G). The Euclidean distance of a motif from the background is simply the Euclidean distance between *M *and *B*, and is given by the following expression:



#### KL distance from background

Let *M *and *B *be as described above. We define the KL distance (Kullback-Leibler distance, also called relative entropy [26]) of a motif from the background as the sum of KL distances between the columns of *M *and *B*:



This is essentially the same evaluation as that used by Frech and coworkers [27].

#### Average fit to motif

Let *s *be a binding site of length *l*. Each such site is associated with a matrix *M *(as above), which describes the consensus distribution over all sites bound by the same transcription factor. We define the fit of *s *to *M *at position *i *as the probability listed in column *i *of matrix *M *for the nucleotide at position *i *of *s*. We define the average fit of *s *to *M *as the average of these values.

#### Probability of site to occur at random

For a binding site *s*, this is simply the product of the probabilities that each nucleotide in *s *will be seen, according to the background distribution.

### Measure of correlation

The data set of 2,928 genes for which binding site information is available was partitioned according to the number of such sites in the gene's promoter region. For each gene, various properties, such as the average length of a binding site in its promoter region, were computed.

We denote as *S*_*i *_the subset of genes with *i *binding sites in their promoter regions. For a given property *P*, we denote its value for a gene *g *by *P*_*g*_, and we define  as follows:



Figures showing correlation of various properties to the number of binding sites depict  as a function of *i *(for example, Figure 3a–d). We note that the variance of the values *P*_*g *_tends to be high in the data set and is not displayed.

To determine whether a property is positively correlated or negatively correlated with the number of binding sites, define for each gene *g *a point (*i*, *P*_*g*_) in the plane, where *i *is the number of binding sites in the promoter region of gene *g*. Let *l*^*obs *^be the linear line that best fits the points (in the sense that it minimizes the sum of squares of the distances). The sign of the slope of *l*^*obs *^defines the correlation as positive or negative.

It should be emphasized that we do not expect a linear relation between the points, and so measuring the Pearson correlation between them is inappropriate. The slope of *l*^*obs *^is simply an *ad hoc *quantifiable measure of whether the correlation is negative or positive.

### Measure of correlation for a specific transcription factor

A similar procedure to that described above is taken when calculating how well binding sites for a specific factor match the overall motif, as a function of the combinatorial regulation in which this factor is involved.

We define the fit of binding site s to a probability matrix *M *describing the corresponding motif as above. The fit of *s *to *M *at position *i *is the probability listed in column *i *of matrix *M *for the nucleotide at position *i *of *s*. The overall fit of *s *to *M*, denoted *f*_*s*_, is the product of these probabilities. In other words, it is the probability that such a sequence will be generated according to the probability matrix *M*.

Let *T *be some transcription factor, and let *R *be the set of promoter regions to which *T *binds. Partition *R *according to the number of binding sites in the promoter (for any factor). Let *R*_*i *_be the subset of promoter regions with *i *binding sites, and let *S*_*i *_be the set of all binding sites for *T *that appear in some promoter region in *R*_*i*_. The average fit of binding sites associated with *T *over promoter regions with *i *binding sites is given by the following equation:



Figure 2a depicts  as a function of *i *for the transcription factor Reb1.

### Estimating the correlation significance

To estimate the significance of a correlation we use random simulation. In each simulation, the binding sites are shuffled at random while keeping the number of sites within each promoter region the same as in the true data. That is, the binding sites map is reordered according to a random permutation. For each gene *g*, the value of the relevant property  (for example, average binding-site length) is then recalculated from the shuffled sites. The random values are used to derive a set of points (*I*, ), as above, and a linear line *l*^rand ^that best fits these points is constructed.

Repeating this simulation *n *times gives us an estimate of the mean value of *l*^rand ^and its standard deviation. In the results reported here, *n *= 10^5^, and for all of the examined scenarios none of the random slopes was as steep as the observed one.

When estimating the significance of the correlation between combinatorial regulation and whether a gene is essential (Figure 3b), the tagging of the genes (essential/nonessential) was shuffled, rather than the binding sites.

Similar simulations were used to estimate the significance of correlation to the number of transcription factors. In doing so, the genes are partitioned according to the number of factors that bind their promoter regions, rather than the number of sites, and the analysis was carried out in the same way as described above.

### Alternative measures for combinatorial regulation

In the analysis discussed, the total number of binding sites, regardless of whether they correspond to the same transcription factor or to different ones, was used as a measure of combinatorial control. We repeated the analysis using the number of transcription factors that bind the promoter region, rather than the total number of binding sites, for this purpose (Additional data files 3 [panel a] and 4). Moreover, the analysis was also repeated on two restricted subsets of promoters: for one, in each promoter all binding sites are associated with the same transcription factor (Additional data files 3 [panel b] and 5); and for the other, in each promoter each binding site is associated with a different factor (Additional data files 3 [panel c] and 6). Although these three scenarios probably represent different definitions for combinatorial control, similar results were obtained in nearly all cases.

## Additional data files

The following additional data are included with the online version of this article: a figure depicting the effective length and fuzziness of motifs as a function of the number of binding sites in the promoter region (Additional data file 1); a figure depicting the correlation between fit of binding sites to the motif and the length of the motif (Additional data file 2); a figure depicting the distribution of promoters according to the number of associated transcription factors/binding sites (Additional data file 3); a figure depicting average promoter and gene properties as a function of the number of transcription factors (Additional data file 4); a figure depicting average promoter and gene properties as a function of the number of binding sites, for promoters to which exactly one factor binds (Additional data file 5); a figure depicting average promoter and gene properties as a function of the number of binding sites, for promoters for which each factor has exactly one binding site (Additional data file 6); and a figure depicting the distribution of correlations between motif length and number of binding sites in randomly shuffled data (Additional data file 7).

## Supplementary Material

Additional data file 1 A figure depicting the effective length and fuzziness of motifs as a function of the number of binding sites in the promoter region Click here for file

Additional data file 2 A figure depicting the correlation between fit of binding sites to the motif and the length of the motif Click here for file

Additional data file 3 A figure depicting the distribution of promoters according to the number of associated transcription factors/binding sites Click here for file

Additional data file 4 A figure depicting average promoter and gene properties as a function of the number of transcription factors Click here for file

Additional data file 5 A figure depicting average promoter and gene properties as a function of the number of binding sites, for promoters to which exactly one factor binds Click here for file

Additional data file 6 A figure depicting average promoter and gene properties as a function of the number of binding sites, for promoters for which each factor has exactly one binding site Click here for file

Additional data file 7 A figure depicting the distribution of correlations between motif length and number of binding sites in randomly shuffled data Click here for file

## Figures and Tables

**Table 1:** Average number of binding sites for genes leading to slow and fast growth

Medium	Low growth rate		High growth rate		*P*
		
	Average number of sites	Number of genes	Average number of sites	Number of genes	
YPD	1.3563	174	1.9530	3642	6.8 × 10^-5^
YPDGE	1.3133	233	1.9588	3569	1.2 × 10^-5^
YPG	1.4188	351	1.9844	3521	9.1 × 10^-7^
YPE	1.4108	370	1.9951	3447	3 × 10^-7^
YPL	1.4114	333	1.9832	3572	5.6 × 10^-7^

The overall average is 1.87. Media: YPD, 2% glucose; YPDGE, 0.1% glucose, 3% glycerol, and 2% ethanol; YPE, 2% ethanol; YPG, 3% glycerol; and YPL, 2% lactate.

**Table 2:** Average number of binding sites according to GO annotations

GO term	Number of genes	Average number of sites	*P*
Metabolism	511	2.02	0.02
Transferase activity	263	2.07	0.06
Amino acid metabolism	68	2.04	0.22
RNA metabolism	65	2.02	0.24
DNA metabolism	60	2.17	0.17
Binding	103	1.22	0.05
Protein modification	49	1.39	0.25

The overall average is 1.72. GO, Gene Ontology.
