# Supplementary material for: The design of transcription-factor binding sites is affected by combinatorial regulation
Source: Genome Biol. 2005 Dec 2;6(12):R103. doi: 10.1186/gb-2005-6-12-r103 (PMC1414079; doi:10.1186/gb-2005-6-12-r103)
Supplement: Additional data file 1 — A figure depicting the effective length and fuzziness of motifs as a function of the number of binding sites in the promoter region [file gb-2005-6-12-r103-S1.pdf]

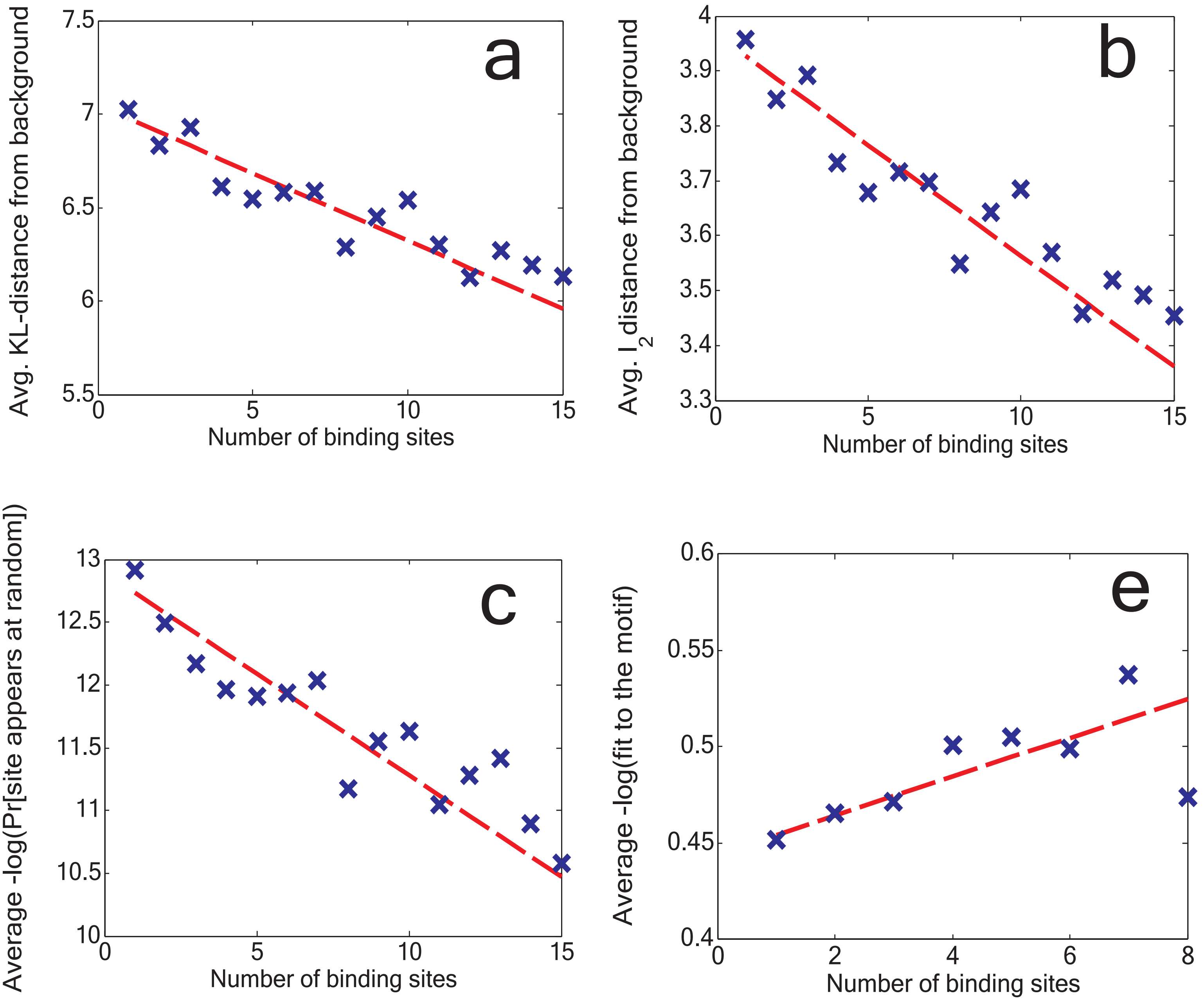

Supplementary Figure 1: Effective length and fuzziness of motifs as a function of the number of binding sites in the promoter region. (a) Average KL-distance of motif from the background distribution. (b) Average Euclidean distance of motif from the background distribution. (c) Average probability that a site appears at random (-log values are shown). (d) Average fit of site to to motif (-log values are shown).

Dashed red lines depict the linear line which best matches the data points.
