# Supplementary material for: The design of transcription-factor binding sites is affected by combinatorial regulation
Source: Genome Biol. 2005 Dec 2;6(12):R103. doi: 10.1186/gb-2005-6-12-r103 (PMC1414079; doi:10.1186/gb-2005-6-12-r103)
Supplement: Additional data file 2 — A figure depicting the correlation between fit of binding sites to the motif and the length of the motif [file gb-2005-6-12-r103-S2.pdf]

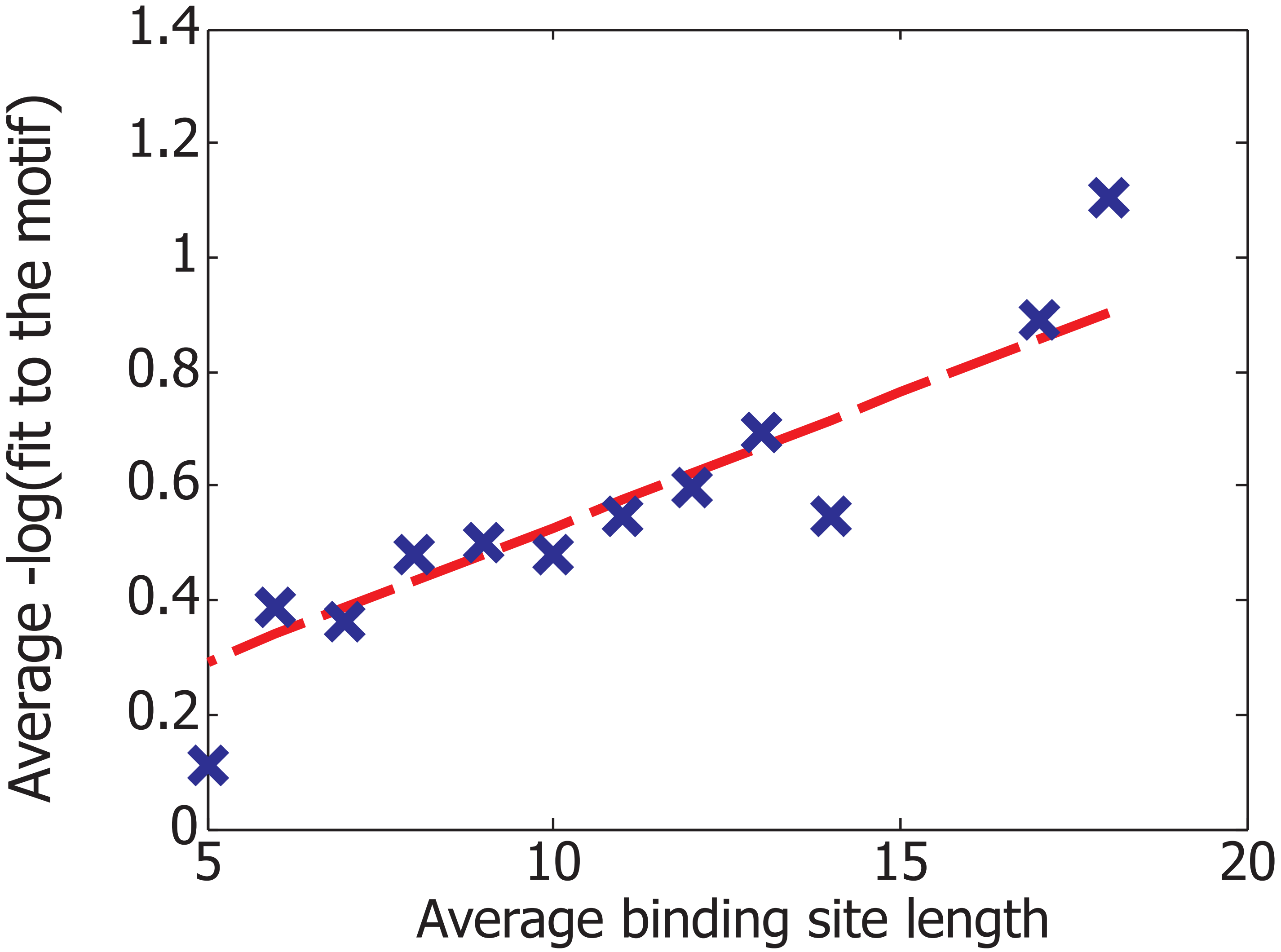

Supplementary Figure 2: Correlation between fit of binding sites to the motif and the length of the motif. For motif fit, average -log values are shown. Dashed red line depicts the linear line which best fits the data.
