# Supplementary material for: The design of transcription-factor binding sites is affected by combinatorial regulation
Source: Genome Biol. 2005 Dec 2;6(12):R103. doi: 10.1186/gb-2005-6-12-r103 (PMC1414079; doi:10.1186/gb-2005-6-12-r103)
Supplement: Additional data file 3 — A figure depicting the distribution of promoters according to the number of associated transcription factors/binding sites [file gb-2005-6-12-r103-S3.pdf]

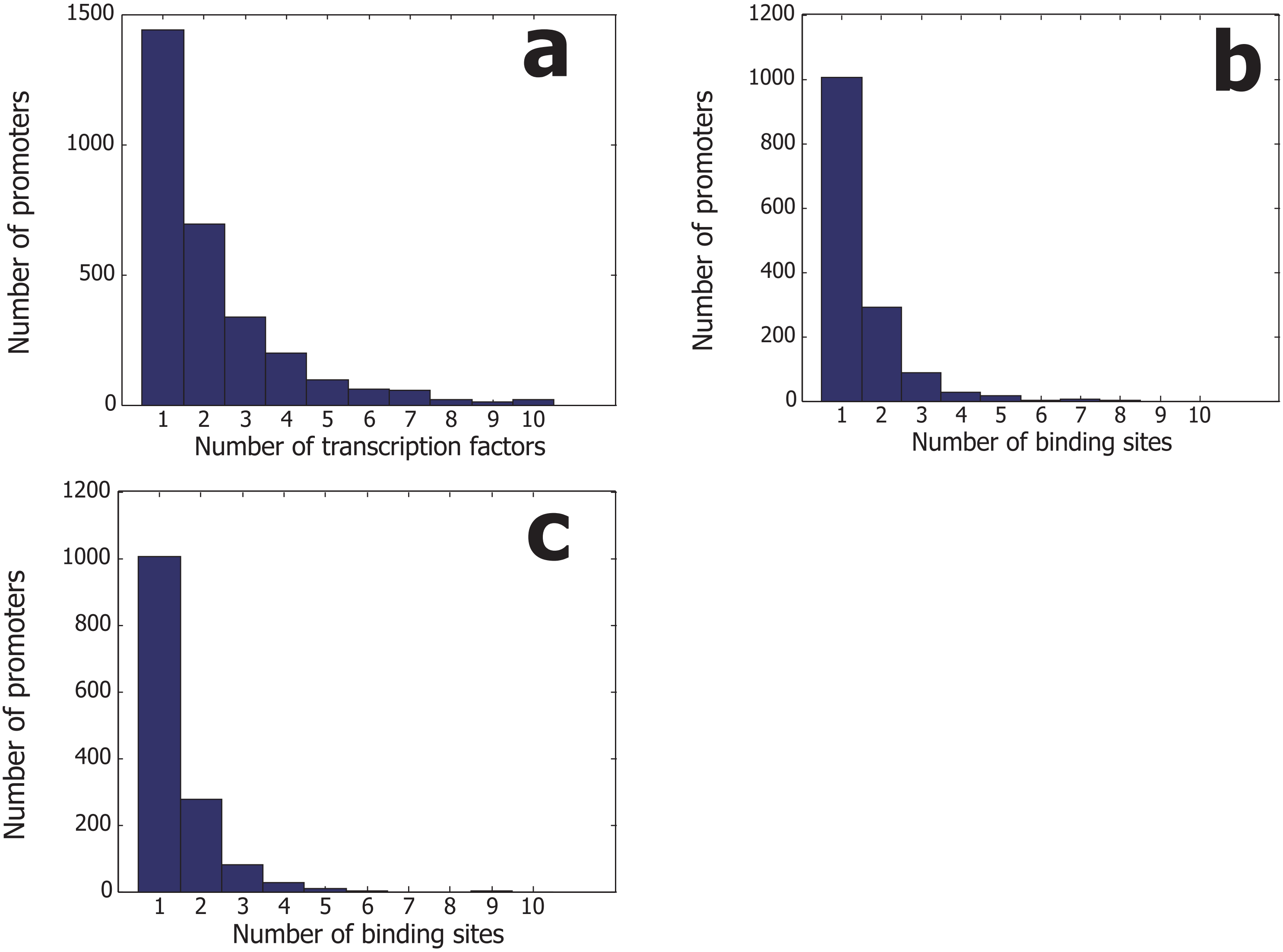

Supplementary Figure 3: Distribution of promoters according to the number of associated transcription factors/binding sites. (a) Distribution of the number of promoters by the number of factors that bind them; (b) Distribution of promoters to which only exactly factor binds by the number of binding sites they contain; (c) Distribution of promoters in which each factor has exactly one binding site by the number of binding sites.
