# Supplementary material for: The design of transcription-factor binding sites is affected by combinatorial regulation
Source: Genome Biol. 2005 Dec 2;6(12):R103. doi: 10.1186/gb-2005-6-12-r103 (PMC1414079; doi:10.1186/gb-2005-6-12-r103)
Supplement: Additional data file 4 — A figure depicting average promoter and gene properties as a function of the number of transcription factors [file gb-2005-6-12-r103-S4.pdf]

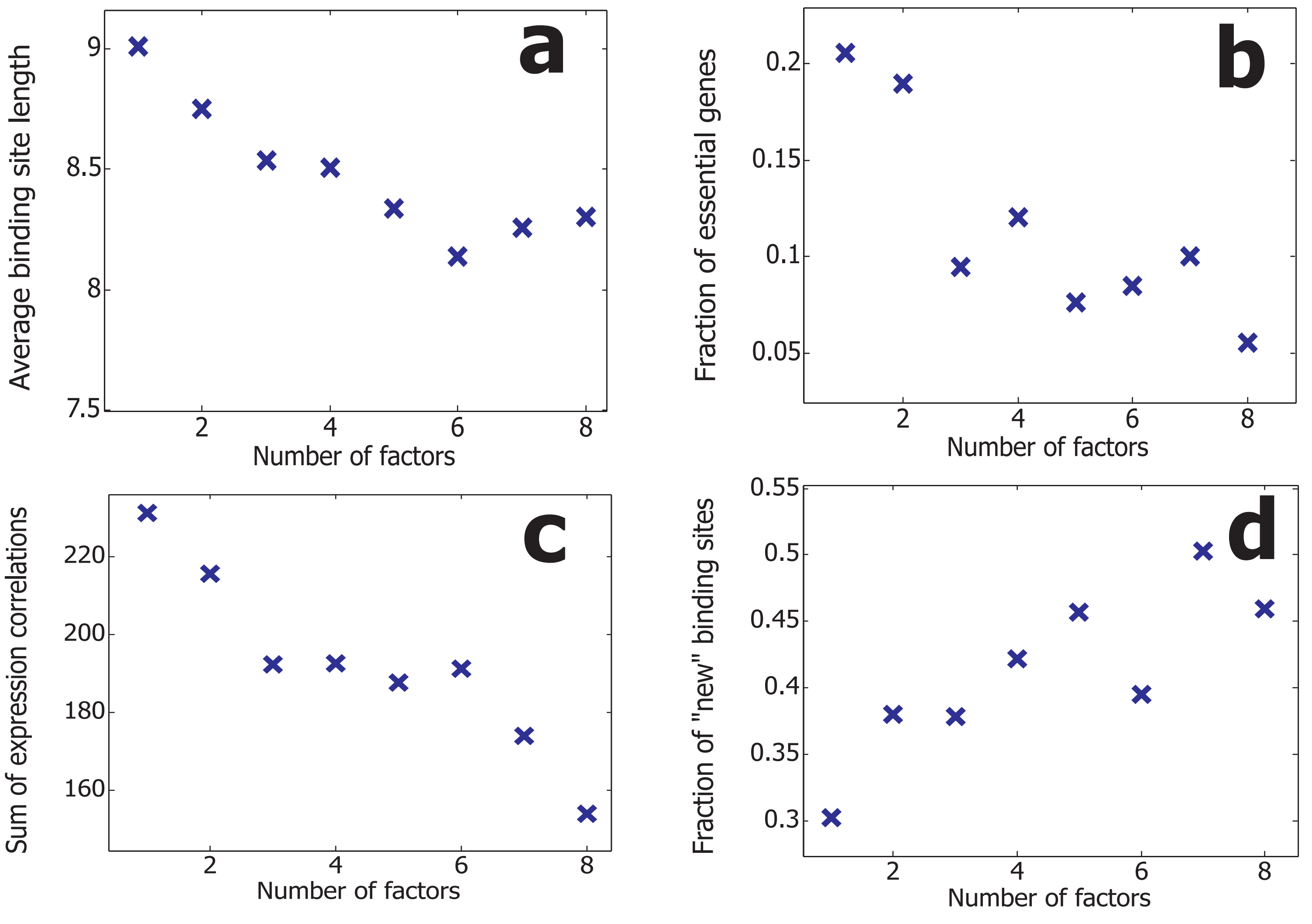

Supplementary Figure 4: Average promoter and gene properties as a function of the number of transcription factors. (a) Average binding site length; (b) Fraction of essential genes; (c) Sum of expression correlations; (d) Fraction of binding sites which are “new” - not conserved in other species.

Graphs show promoters to which up to 8 transciption factors bind. These constitute 99% of the promoters to which at least one transcription factor binds.
