# Supplementary material for: The design of transcription-factor binding sites is affected by combinatorial regulation
Source: Genome Biol. 2005 Dec 2;6(12):R103. doi: 10.1186/gb-2005-6-12-r103 (PMC1414079; doi:10.1186/gb-2005-6-12-r103)
Supplement: Additional data file 6 — A figure depicting average promoter and gene properties as a function of the number of binding sites, for promoters for which each factor has exactly one binding site [file gb-2005-6-12-r103-S6.pdf]

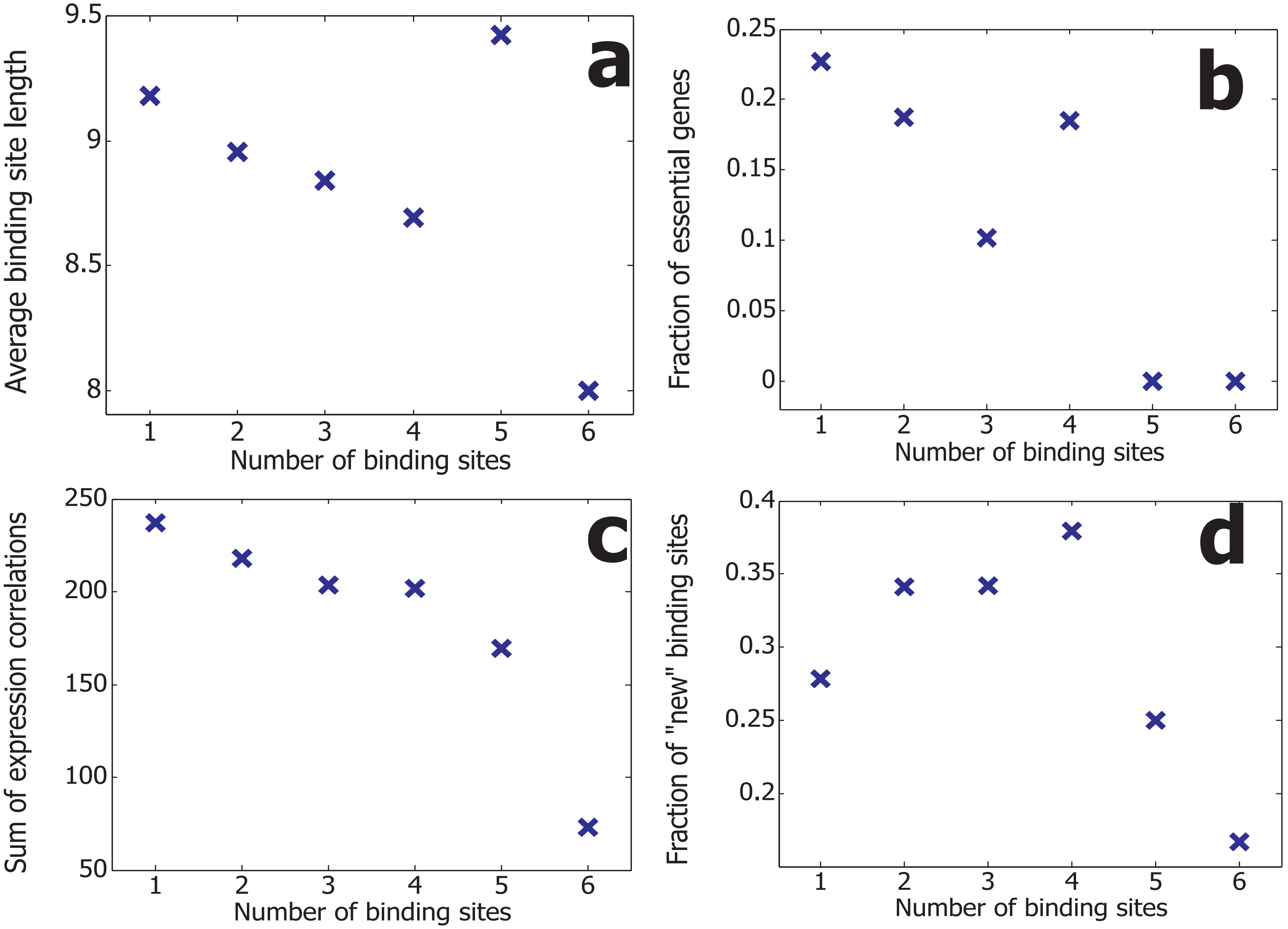

Supplementary Figure 6: Average promoter and gene properties as a function of the number of binding sites, for promoters where each factor has exactly 1 binding site. (a) Average binding site length; (b) Fraction of essential genes; (c) Sum of expression correlations; (d) Fraction of binding sites which are “new” - not conserved in other species.
