# Supplementary material for: The design of transcription-factor binding sites is affected by combinatorial regulation
Source: Genome Biol. 2005 Dec 2;6(12):R103. doi: 10.1186/gb-2005-6-12-r103 (PMC1414079; doi:10.1186/gb-2005-6-12-r103)
Supplement: Additional data file 7 — A figure depicting the distribution of correlations between motif length and number of binding sites in randomly shuffled data [file gb-2005-6-12-r103-S7.pdf]

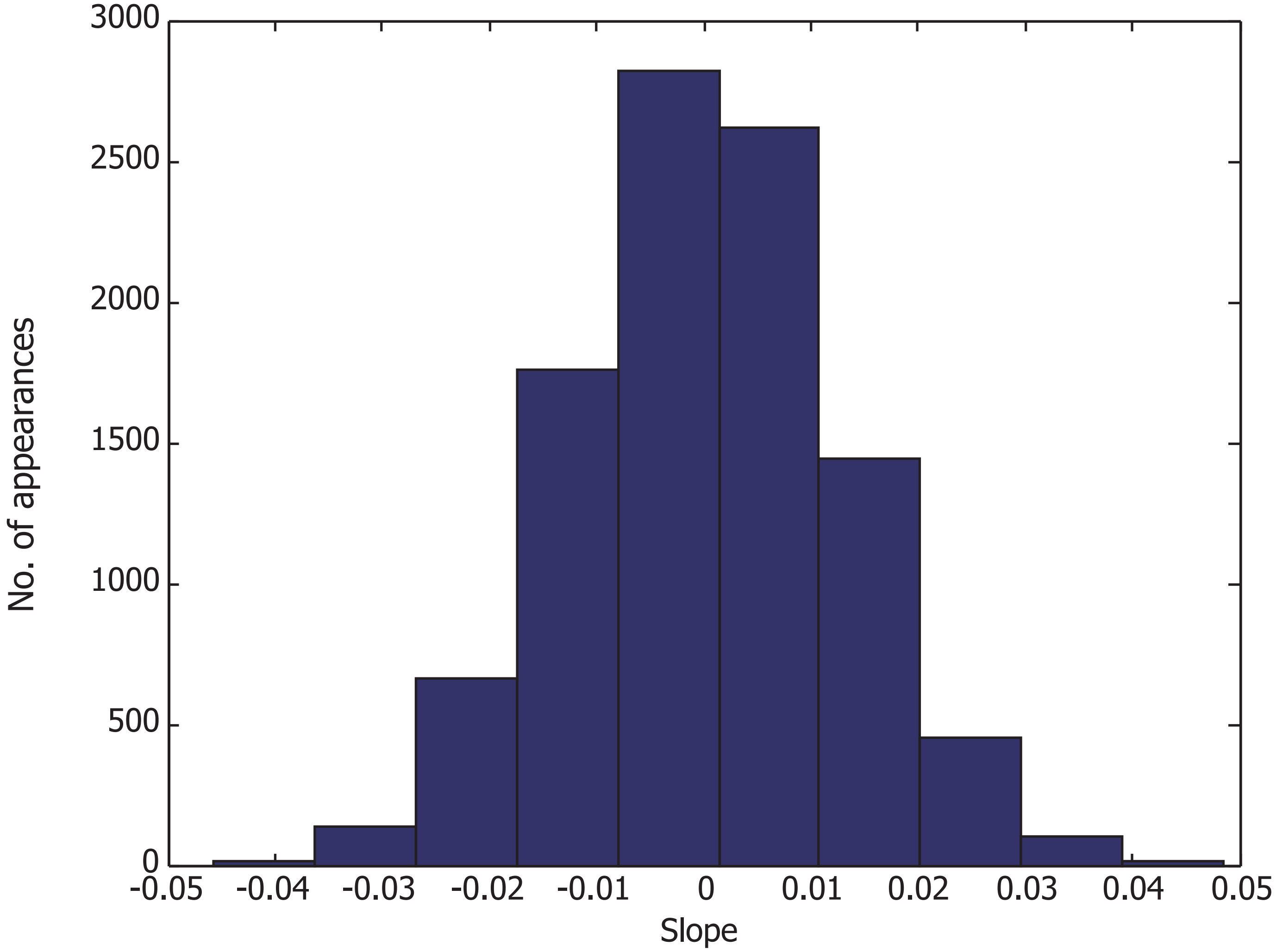

Supplementary Figure 7: Distribution of correlations in shuffled data. The distribution of slopes in shuffled data, of lines fitting average length of motif vs. number of binding sites (as in fig. 3a). Value in real data is -0.1109. The figure depicts 10000 simulations.
